# CRISPR/CAS9 mutagenesis of a single *r-opsin* gene blocks phototaxis in a marine larva

**DOI:** 10.1098/rspb.2018.2491

**Published:** 2019-06-05

**Authors:** S. Neal, D. M. de Jong, E. C. Seaver

**Affiliations:** Whitney Laboratory for Marine Bioscience, University of Florida, St Augustine, FL, 32080 USA

**Keywords:** *opsin*, phototaxis, *Capitella teleta*, annelid, behaviour, genome editing

## Abstract

Many marine animals depend upon a larval phase of their life cycle to locate suitable habitat, and larvae use light detection to influence swimming behaviour and dispersal. Light detection is mediated by the *opsin* genes, which encode light-sensitive transmembrane proteins. Previous studies suggest that *r-opsins* in the eyes mediate locomotory behaviour in marine protostomes, but few have provided direct evidence through gene mutagenesis. Larvae of the marine annelid *Capitella teleta* have simple eyespots and are positively phototactic, although the molecular components that mediate this behaviour are unknown. Here, we characterize the spatio-temporal expression of the rhabdomeric *opsin* genes in *C. teleta* and show that a single rhabdomeric *opsin* gene, *Ct-r-opsin1*, is expressed in the larval photoreceptor cells. To investigate its function, *Ct-r-opsin1* was disrupted using CRISPR/CAS9 mutagenesis. Polymerase chain reaction amplification and DNA sequencing demonstrated efficient editing of the *Ct-r-opsin1* locus. In addition, the pattern of *Ct-r-opsin1* expression in photoreceptor cells was altered. Notably, there was a significant decrease in larval phototaxis, although the eyespot photoreceptor cell and associated pigment cell formed normally and persisted in *Ct-r-opsin1*-mutant animals. The loss of phototaxis owing to mutations in *Ct-r-opsin1* is similar to that observed when the entire photoreceptor and pigment cell are deleted, demonstrating that a single *r-opsin* gene is sufficient to mediate phototaxis in *C. teleta*. These results establish the feasibility of gene editing in animals like *C. teleta*, and extend previous work on the development, evolution and function of the *C. teleta* visual system*.* Our study represents one example of disruption of animal behaviour by gene editing through CRISPR/CAS9 mutagenesis, and has broad implications for performing genome editing studies in a wide variety of other understudied animals.

## 1. Introduction

Many marine animals have a larval dispersal phase to locate and move towards a suitable habitat. For marine larvae, eyes often provide information about light intensity and direction, and are thought to mediate the positive or negative phototactic responses that are important for both dispersal and settlement [1,2]. The majority of pelagic larvae produced by benthic marine invertebrates have a period of positive photo response [1]. Larvae typically have simple eyes that can be comprised of only two cells: a pigment cell and a photosensory cell [2–5]. The pigment cell shields incoming light, and its close proximity to the photosensory cell is sufficient for detection of the direction of light [3].

**Figure 1.**
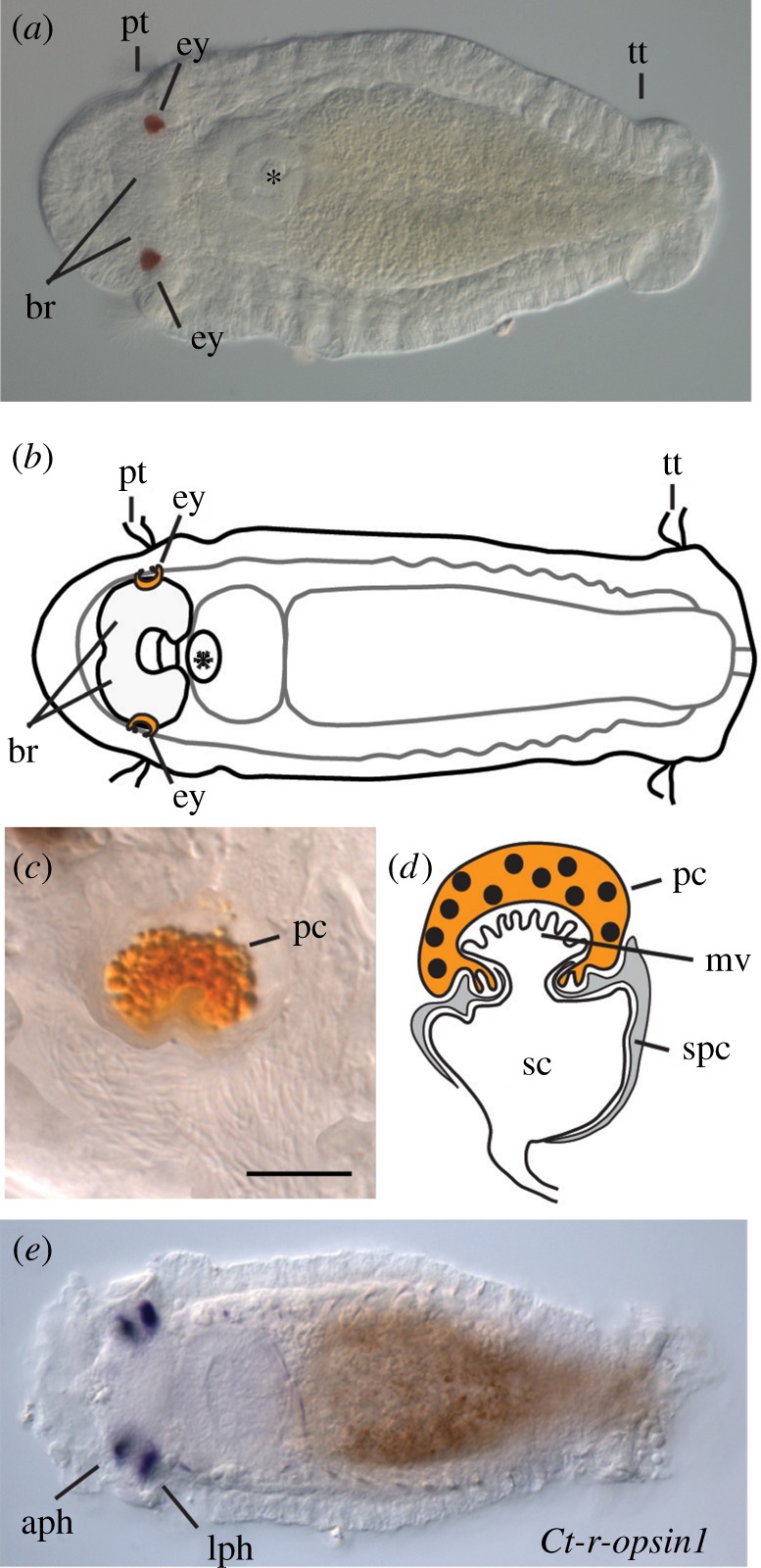
*Capitella* larval brain and eyespot structure and *Ct-r-opsin1* expression. (*a*) *Capitella teleta* larva. (*b*) Schematic of (*a*). (*c*) Enlarged image of right larval eye showing pigment granules in the pigment cell. Scale bar, 5 µm. (*d*) Schematic of larval eye showing sensory, pigment and supporting cells. Adapted from [8]. (*e*) *In situ* hybridization showing mRNA expression of *Ct-r-opsin1*. (*a*, *b* and *e*) are ventral views of a stage 7 larva. Anterior is to the left. aph, adult photoreceptor; br, brain; ey, eye; lph, larval photoreceptor; mv, microvilli; pc, pigment cell; sc, sensory cell; spc, supporting cell; pt, prototroch; tt, telotoch. (Online version in colour.)

*Capitella teleta* is an annelid worm that burrows in marine sediments and produces a swimming larva as part of its life cycle [6]. *Capitella teleta* larvae have a pair of eyespots similar to the simple larval eyespots characteristic of many invertebrate larvae, and similar to the prototype pigment-cup eye proposed to represent the ancestral bilaterian condition [3,7]. The larval eyespot in *C. teleta* is located along the exterior rim of the brain, and is composed of a supporting cell, photosensory cell and pigment cell (figure 1*a–d*; [8]). The eyespots appear soon after initiation of the larval period, prior to robust swimming [9]. The juvenile photosensory cell appears during late larval stages and temporally coexists with the larval eyespot [10]. During metamorphosis, the larval pigment cell is incorporated into the juvenile eyespot [10], although larval and juvenile photosensory cells appear to be distinct. *Capitella teleta* larvae exhibit a robust positive phototactic response (electronic supplementary material, figure S1). This behaviour is lost if both photoreceptor and pigment cells are experimentally deleted, demonstrating that phototaxis is mediated by the cerebral eyespots [10,11].

Light detection and processing in the photoreceptor cells are mediated by members of the *opsin* gene family [12], a large monophyletic subclass within the G-protein coupled receptor superfamily [13]. Opsin proteins contain a seven-pass transmembrane domain and a G-protein coupled receptor domain [14]. Different classes of *opsin* genes are generally associated with distinct photoreceptor cell types, which are distinguished by their apical cell membrane morphology [12]. That is, ciliary photoreceptors express ciliary *opsin* genes and rhabdomeric photoreceptors express rhabdomeric *opsin* genes. The cerebral eyes in larval and adult polychaetes typically have rhabdomeric photoreceptor cells, although there are exceptions [3,4,15]. In the last common ancestor of bilaterians, nine classes of *opsin* genes were thought to have been present [16]. The genome of *C. teleta* contains nine *opsin* genes that belong to only two *opsin* classes: three rhabdomeric *opsin* (*r-opsin)* and six *neuropsin* genes [16,17]. Notably, *C. teleta* lacks ciliary *opsin* genes; a similar situation is found in most other lophotrochozoans [16].

Although the evolution and expression of *opsin* genes has been characterized in many taxa, few studies have demonstrated a functional role for *opsin* genes in marine larvae. Here, we explore the function of *opsin* genes in mediating larval phototactic behaviour of *C. teleta*. The availability of a sequenced genome [18], a comprehensive embryonic fate map [19], and the availability of a breeding laboratory colony make *C. teleta* a valuable system for studies of development and evolution within the lophotrochozoan clade. We characterize expression of all of the rhabdomeric *opsin* genes and three *neuropsin* genes in larvae by *in situ* hybridization. Using CRISPR/CAS9 mutagenesis, we investigate the function of *Ct-r-opsin1*, the only *opsin* gene expressed in the larval photosensory cell. Through direct genotyping, *in situ* hybridization and behavioural analysis, we demonstrate that *Ct-r-opsin1* is sufficient to mediate positive phototaxis. In addition, we establish CRISPR/CAS9 mutagenesis as an efficient method for studies of gene function in *C. teleta*.

## 2. Results

### (a) Opsin expression in *Capitella teleta*

**Figure 2.**
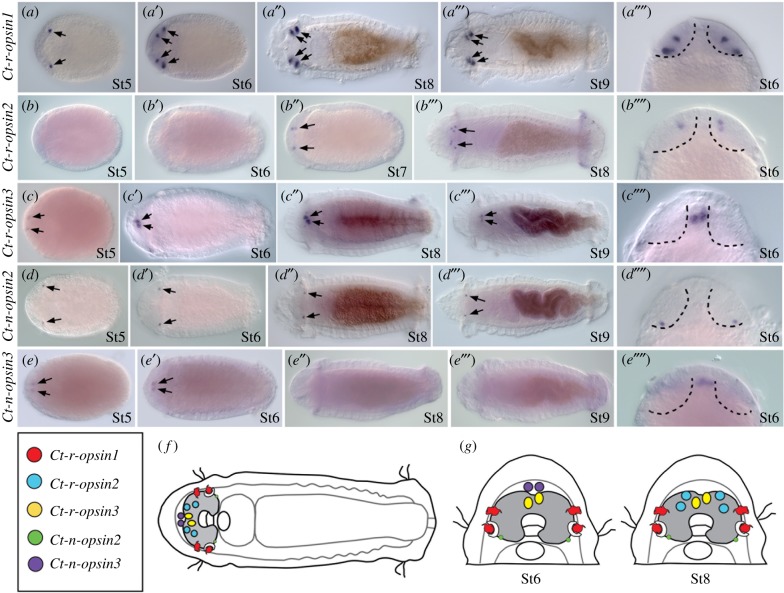
Expression of *opsin* genes in the larval brain region and eyespot sensory cells. (*a* – *e′′′′*) Expression patterns of *opsin* genes by *in situ* hybridization during larval development. Arrows indicate expression. All panels are ventral views. Stages are indicated in bottom right corner and follow the staging system of [20]. (*a*–*e′′′*) Anterior is to the left. (*a′′′′*–*e′′′′*) Enlarged views of head. Brain lobes are indicated by dotted lines. Anterior is to the top. Brain is shaded in (*f*) and (*g*). (*a*–*a′′′′*) *Ct-r-opsin1*, (*b*–*b′′′′*) *Ct-r-opsin2*, (*c*–*c′′′′*) *Ct-r-opsin3*, (*d*–*d′′′′*) *Ct-n-opsin2*, (*e*–*e′′′′*) *Ct-n-opsin3*. (*f*) Schematic of a stage 7 larva, showing localization of *opsin* expression domains to the head. Each gene is represented by a distinct colour (see key). (*g*) Schematics showing magnified views of larval heads (stages 6 and 8) to highlight the spatial relationships among *opsin* gene expression patterns. (Online version in colour.)

We characterized expression of all of the rhabdomeric *opsin* genes and three of the *neuropsin* genes present in the *C. teleta* genome. We analysed these expression patterns during larval development by *in situ* hybridization (figure 2). Our rationale for focusing on the rhabdomeric *opsin* genes is that the photoreceptor cells in *C. teleta* larval eyespots were previously shown to be the rhabdomeric type [8,10], and rhabdomeric *opsin* genes typically mediate photodetection and vision in protostomes [2,21,22]. One *neuropsin* gene, *Ct-n-opsin1*, was undetectable at the stages examined, even with varying conditions (data not shown).

Each *opsin* gene investigated shows a unique expression pattern, and transcripts of all five genes are restricted to 2–6 cells (figure 2). *Ct-r-opsin1* is expressed in both the larval and adult photosensory cells (figures 1*e* and 2*a*–*a′′′′*). The adult photosensory cells are located anterior to the larval eyespots (figure 2*a′*–*a′′′′*) [10]. *Ct-r-opsin2* is expressed in a small number of cells in the brain region of late larval stages, but is not detectable at early stages (figure 2*b*–*b′′′′*). *Ct-r-opsin3* is expressed in a pair of medial cells throughout larval stages (figure 2*c*–*c′′′′*). *Ct-n-opsin2* also shows a stable expression pattern across larval stages and is detected in a pair of lateral cells (figure 2*d*–*d′′′′*). *Ct-n-opsin3* is only detectable in early larval stages in a pair of cells medial to the brain lobes (figure 2*e*–*e′′′′*). Of these, only *Ct-r-opsin1* is expressed in the photosensory cell of the eyespot. *Ct-r-opsin1* is present as the eyespots form, during the period of larval phototaxis, and in juvenile eyespots (not shown). In summary, transcripts of the five *opsin* genes are localized to the head, have unique patterns and are closely associated with the brain (figure 2*f,g*).

### (b) CRISPR/CAS9-mediated genome editing of *Ct-r-opsin1* is highly efficient

**Figure 3.**
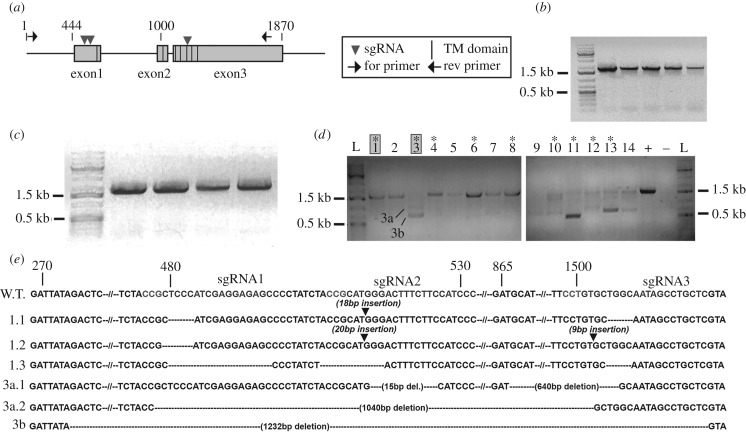
CRISPR/CAS9-mediated knockout of *Ct-r-opsin1*. (*a*) *Ct-r-opsin1* genomic locus showing sgRNA target sites (arrowheads), primer binding sites (black arrows), intron-exon structure (grey shaded boxes are exons, intervening black lines are introns), and the seven-transmembrane (TM) domains [11] (black vertical lines within exons). (*b*,*c*) Gel electrophoresis showing amplicons of the *Ct-r-opsin1* locus from control animals injected with CAS9 protein only (*b*) or sgRNA only (*c*). Primer positions are indicated in (*a*). Each lane represents a PCR product of DNA extracted from an individual larva. (*d*) Amplicons of the *Ct-r-opsin1* locus from 14 individual experimental larvae (1–14) using primers indicated in (*a*). Larvae resulted from embryos injected with sgRNA/CAS9 complexes. Asterisks indicate lanes from whom bands were cloned and sequenced. Grey shading of lanes 1 and 3 indicates larvae from which sequencing results are shown in figure 3*e*. For larva 1, the single wild-type-sized band was cloned and multiple clones were sequenced. For larva 3, bands 3a and 3b were cloned separately, and multiple clones from each sequenced. L, ladder. 0.5 kb and 1.5 kb bands of the ladder are marked.+ indicates positive control in which PCR was conducted on gDNA extracted from a wild-type larva, and − indicates the negative (no template) control. (e) Genome sequences showing indels. Wild-type sequence is in the top row (W.T.). Position of target sequences are indicated above sequence. Numbers indicate genomic position within the locus (figure 3*a*). --//-- represents a large section of sequence that is not shown. Sequences of three individual clones derived from larva 1 are shown as 1.1, 1.2 and 1.3, and sequences of three clones derived from larva 3 are shown as 3a.1, 3a.2 and 3b. Dashes indicate deletions; black arrowheads indicate insertions.

To test the function of *Ct-r-opsin1* in *C. teleta* larvae, we generated a *Ct-r-opsin1* mutant using CRISPR/CAS9 gene editing. *Ct-r-opsin1* is encoded by three exons (figure 3*a*). *Opsin* genes typically have seven transmembrane domains, and they are spread across all three exons in the *Ct-r-opsin1* gene. We designed three single guide RNAs (sgRNAs) directed against *Ct-r-opsin1*, two that target sites in the first exon, and a third that targets a site towards the 5′ end of exon 3 (figure 3*a*). Fertilized single cell zygotes were microinjected with CAS9 protein/sgRNA complexes containing all three sgRNAs in a single injection cocktail, and *F*_0_ stage 7 larvae resulting from these injections were analysed. We examined three different conditions marked by differing ratios of CAS9 : sgRNA (1 : 1, 1.2 : 1, 1.7 : 1) by polymerase chain reaction (PCR) analysis, *in situ* hybridization, and a phototactic assay (electronic supplementary material, table S1). Additionally, we sequenced DNA extracted from larvae that were injected with the 1.2 : 1 CAS9 : sgRNA ratio.

We analysed genome editing events in individual larvae by PCR screening of genomic DNA (figure 3*d*) and DNA sequencing (figure 3*e*). CAS9 only and sgRNA only controls displayed an expected amplicon size following PCR analysis (figure 3*b* and *c*, respectively). Of the 34 experimental larvae analysed by PCR analysis, 12 larvae had wild-type-sized amplicons (electronic supplementary material, table S1). Figure 3*d* shows banding patterns of 14 examples from the 34 experimental larvae analysed by PCR analysis. A subset of wild-type and non-wild-type-sized bands from nine experimental individuals were cloned and sequenced (figure 3*d*, asterisks). All nine larvae had at least one clone with a mutation in the *Ct-r-opsin1* gene (total of 53 sequenced clones), and only 3 out of 53 clones displayed a wild-type sequence (electronic supplementary material, table S1). Although bands from four of the sequenced larvae had only wild-type amplicon sizes, only 3 out of 19 clones sequenced from these larvae contained wild-type sequences, indicating that clones appearing to be wild-type by PCR analysis actually contained small indels. Therefore, PCR analysis underestimates the efficiency of CRISPR/CAS9 mutagenesis, because PCR analysis was unable to distinguish small deletions/insertions from wild-type-sized amplicons.

We observed both large and small deletions, and multiple unique cutting events per individual. By analysing single larvae, we could detect the presence of multiple distinct indels (figure 3*e*). In one example (larva 1), clones isolated from a wild-type-sized amplicon show numerous small indels (figure 3*e*, clones 1.1, 1.2, 1.3). Sequence 1.1 includes two small deletions at the sites targeted by sgRNA1 (4 bp) and sgRNA3 (4 bp), and there is an 18 bp insertion at the site of sgRNA2 (figure 3*e*). Therefore, all three guide RNAs caused mutations, and these resulted in frameshifts in the reading frame. Another example comes from larva 3 whose *r-opsin1* locus has large-scale deletions (figure 3*e*; larva 3, clones 3a.1, 3a.2 and 3b). Clone 3a.1 contains two deletions in the sequence targeted by sgRNA2 and sgRNA3, whereas clone 3a.2 contains a 1040 bp deletion spanning the regions targeted by sgRNA1 and sgRNA3. Clone 3b contains a large deletion originating approximately 200 bp 5′ of the sequence targeted by sgRNA1 and extends to the sequence targeted by sgRNA3 (figure 3*e*). Either a frameshift mutation or large deletion will result in a truncated protein that will probably not localize to the membrane, and therefore be non-functional. Our observations of distinct cutting events within an individual demonstrates the mosaic nature of the genomic mutations.

### (c) Effect of CAS9 : sgRNA ratio on efficiency of gene editing

**Figure 4.**
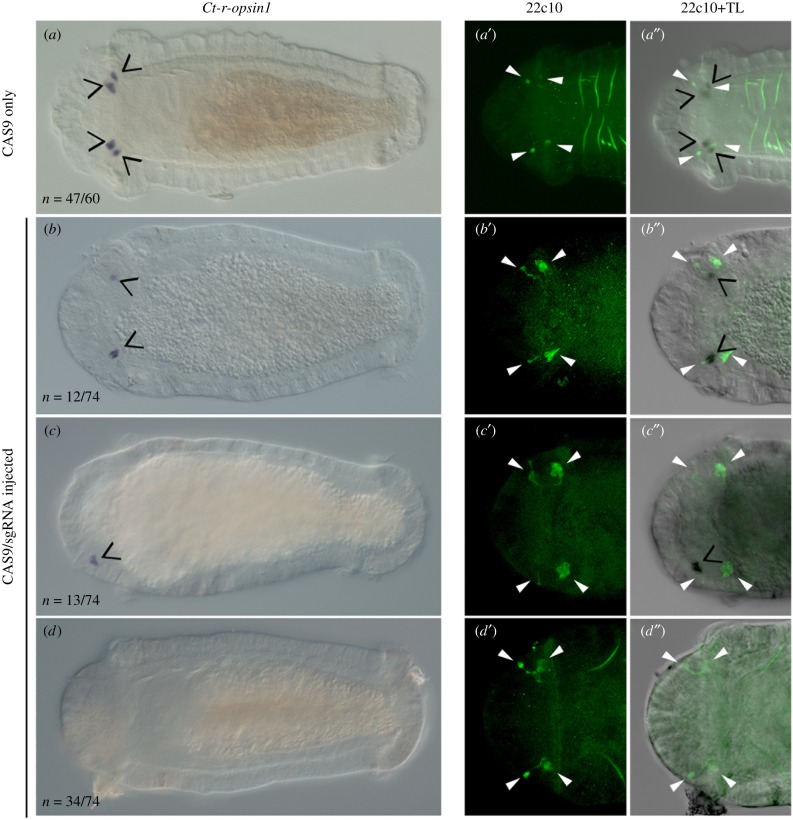
Expression analysis of *Ct-r-opsin1* in mutant larvae. Panels (*a*–*d*) show differential interference contrast microscopy images of *Ct-r-opsin1* expression. The number of larvae displaying the expression pattern shown over the total number of cases examined is indicated in the bottom left corner. (*a*) CAS9-only control animals. (*b*–*d*) Experimental larvae. Panels (*b*–*d*) include larvae injected with the 1.7 : 1 and 1.2 : 1 CAS9 : sgRNA ratio. Panels *a′*, *b′*, *c′* and d′ are confocal stacks showing 22C10 antibody labelling to visualize sensory cells, and panels *a″*, *b″*, *c″*, *d″* show a merge of 22C10 confocal images and transmitted light images. Panels in each row are from a single individual. Open black arrowheads indicate *Ct-r-opsin1* expression, and closed white arrowheads indicate sensory cells.

When we varied the molar ratio of CAS9 protein to sgRNA, there were differences in genome editing efficiency (electronic supplementary material, table S1). Because PCR analysis substantially underestimates genome editing events (see the previous section), we ascertained genome editing efficiency by determining the percentage of larvae with a wild-type expression pattern of the *Ct-r-opsin1* transcript by *in situ* hybridization. The 1 : 1 ratio of CAS9 to sgRNA was the least effective. That is, most larvae resulting from zygote injections with a 1 : 1 ratio had wild-type *Ct-r-opsin1* expression patterns (84%; electronic supplementary material, table S1). Likewise, a high percentage of larvae resulting from zygotes injected with the sgRNA only or CAS9 only controls displayed wild-type expression (90% and 78%, respectively; electronic supplementary material, table S1, figure 4). By contrast, few larvae resulting from injections with CAS9 : sgRNA ratios of either 1.2 : 1 or 1.7 : 1 displayed wild-type *Ct-r-opsin1* expression patterns (6% and 0%, respectively; electronic supplementary material, table S1). In approximately 46% of experimental larvae, *Ct-r-opsin1* expression was not detectable (figure 4*d*). Of the larvae resulting from zygotic injections with the 1.2 : 1 ratio, 8 out of 25 (32%) had no detectable transcript, and of the resulting larvae injected with 1.7 : 1 ratio, 26 out of 49 (53%) had no detectable transcript. Therefore, differences in the ratio between CAS9 and sgRNA in the injectant influenced mutation efficiency, and increasing relative levels of CAS9 produced more robust results.

### (d) Detection of *Ct-r-opsin1* transcript

Multiple distinct expression patterns for *Ct-r-opsin1* were recovered from larvae resulting from zygotic injection of the two most effective CAS9 : sgRNA molar ratios (1.2 : 1 and 1.7 : 1) (figure 4*a*–*d*). Six expression patterns were observed; the *Ct-r-opsin1* transcript was detected in either zero photoreceptors, one photoreceptor, two photoreceptors, three photoreceptors or four photoreceptors. Additionally, in some embryos, although expression was detected in all four photoreceptors, at least one domain was weak relative to the others. This expression pattern was scored as abnormal. We interpret the observed range of expression patterns as a likely sign of mosaicism, or a lack of nonsense-mediated mRNA decay for some, but not all mutations [23].

### (e) Normal eyespot formation in *Ct-r-opsin1* mutants

We examined whether the eyespots form normally in larvae resulting from CAS9/sgRNA injections into zygotes. The monoclonal antibody 22C10 specifically labels photosensory cells in the juvenile and larval eyes in *C. teleta*, and co-localizes with *Ct-r-opsin1* in the photosensory cells [10,24]. In wild-type larvae, *Ct-r-opsin1* mRNA transcript co-localizes with the 22C10 labelling (figure 4*a*–*a′′*). In larvae that have disrupted *Ct-r-opsin1* expression patterns, all four photosensory cells are present (figure 4*b*–*d′′*). Therefore, the photosensory cell of the eyespot develops in the correct location and has axonal processes even in the absence of, or reduction of *Ct-r-opsin1* transcript. The pigment cell of the eyespot is also present in larvae resulting from CAS9/sgRNA injections (not shown).

### (f) *Ct-r-opsin1* knockdown inhibits phototactic behaviour

**Figure 5.**
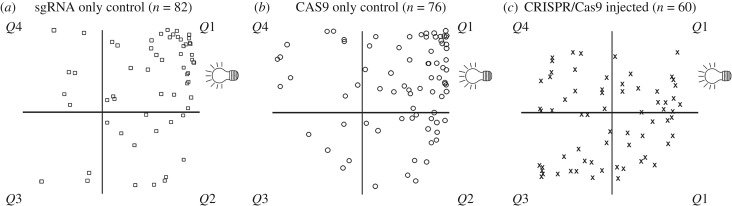
Loss of phototaxis following CRISPR/Cas9-mediated knockout of *Ct-r-opsin1*. Schematics represent the area of the glass cuvette used for the phototaxis assay, divided into four quadrants (*Q*1, *Q*2, *Q*3, *Q*4). Light bulb symbol indicates position of the light source (*Q*1). Shapes indicate positions of individual larvae 20 s after initial light exposure. Control larvae are indicated by squares (sgRNA only) or circles (CAS9 only), and crosses indicate CAS9/sgRNA injected larvae. The number of animals per quadrant after a 20 s light exposure is (*a*) *n* = 44 for *Q*1 and *n* = 38 for *Q*2–4, (*b*) *n* = 43 for *Q*1 and *n* = 33 for *Q*2–4, (*c*) *n* = 17 for *Q*1 and *n* = 43 for *Q*2–4.

To determine whether *Ct-r-opsin1* mediates the phototactic response in *C. teleta* larvae, previously established phototaxis assays [10] were performed with larvae resulting from zygotes injected with CAS9/sgRNA complexes. Phototaxis assays were performed with 10 larvae at a time, and with only 10 larvae in the cuvette, the larvae swim freely. Each set of 10 larvae was considered as an independent biological replicate. All phototaxis assays were performed with a minimum of five independent replicates per subset of embryos. After a 20 s exposure to a point source of light, the position of each larva was recorded. We observed that both sets of control larvae (CAS9 only and sgRNA only), displayed positive phototaxis as exhibited by displacement towards the light source (figure 5*a*,*b*). This behaviour is similar to previous reports of unmanipulated larvae [10]. By contrast, larvae resulting from CAS9/sgRNA injections did not display significant phototaxis (figure 5*c*). The distribution of larvae in the quadrant closest to the light source (*Q*1) relative to the other quadrants was higher in the CAS9 only controls compared with larvae resulting from CAS9/sgRNA injections (Fisher's exact test, *p* = 0.0002; figure 5). The larvae resulting from CAS9/sgRNA injections behaved similarly to larvae in which the photoreceptor and pigment cell were deleted [10]. This indicates that *Ct-r-opsin1* expression in the photoreceptor is sufficient to mediate the robust positive phototactic response in *C. teleta* larvae.

## 3. Discussion

### (a) Comparison of *opsin* expression patterns

*Ct-r-opsin1* is the only *r-opsin* gene expressed in the photosensory cell of the simple eye in *C. teleta* larvae. Although not in the eyespots, the two other *r-opsins*, *r-opsin2* and *r-opsin3*, are also expressed in the cephalic region. *Platynereis dumerilii* is another annelid whose larva exhibits positive phototaxis, and like in *C. teleta*, *r-opsin* is expressed in both larval and adult eyespots [25]. However, unlike the restricted cephalic expression in *C. teleta, r-opsin1* and *r-opsin3* in *P. dumerilii* are also expressed in the segmented trunk [26,27]. Furthermore, *r-opsin1* and *r-opsin3* are expressed in adjacent photoreceptors within the larval eyespot and are co-expressed in the same photoreceptor cells in adult eyes of *P. dumerilii* [27], a contrast with only a single *r-opsin* gene expressed in the photosensory cell of the eyespot in *C. teleta*. There is extraordinary diversity of eye structure, complexity and function in annelids [4]. Studies of *opsin* gene expression and function are one way to understand this diversity and the evolution of light detection in annelids.

Far less is known about the expression and function of *neuropsin* genes relative to *r-opsins*, particularly outside of the vertebrate lineage [16]. Two of the *C. teleta neuropsin* genes, *Ct-n-opsin2* and *Ct-n-opsin3*, are expressed in a small subset of cells in the brain region. The first described *neuropsin*, *Opn5*, is expressed in the brain, spinal cord, eye and testis of mice [28]. *Opn5* has a peak sensitivity to ultraviolet light in chicken [29], mice, and humans [30], and in birds, it is thought to function in seasonal reproduction [31]. Although the *neuropsin* genes are unlikely to be involved in phototaxis, it will be possible to leverage genome editing tools to uncover their function in *C. teleta*.

### (b) Highly efficient CRISPR/CAS9-mediated genome editing in *Capitella teleta*

The results of this study dramatically improve preliminary attempts at genome editing [6], and demonstrate that CRISPR/CAS9 mediated genome editing is an effective method for generating targeted mutations in *C. teleta*. Sequence analysis and analysis by *in situ* hybridization indicate a mutation rate of 94% and 100%, respectively. By contrast, PCR analysis underestimates CRISPR/CAS9-mediated genome editing because it does not detect small indels. Therefore, it is important to carefully choose the detection method for CRISPR/CAS9-induced mutation. Owing to the efficient mutation rate, we can evaluate phenotypes in the *F*_0_ generation [32].

We think that our high rate of genome editing was achieved by using three sgRNAs targeted to *Ct-r-opsin1* along with injection of CAS9 protein. Injection of three sgRNAs together is highly efficient in generating mutants in other animals [33]. We observed mutations associated with all three sgRNA target sites, and we recovered both large and small deletions. Additionally, microinjecting CAS9 protein has been shown to be substantially more efficient than injecting Cas9 mRNA, and may decrease mosaicism [34,35]. We observed an increased frequency of mutation associated with increasing CAS9 protein levels relative to sgRNA in the injectant. Increasing CAS9 protein concentration may lead to more efficient complex formation *in vitro*, and in turn, more efficient *in vivo* genomic editing. Although gene editing was efficient, we did detect mosaicism. This may be explained by DNA editing events that occurred after the zygote cleaved into multiple cells.

### (c) Behavioural adaptations to the marine environment

Our results demonstrate that *Ct-r-opsin1* is sufficient to mediate positive phototaxis. The *Ct-r-opsin1* mutants behaved similarly to animals in which the photoreceptor and pigment cell are experimentally deleted [10]. It is important to note that in our *Ct-r-opsin1* mutants, both the pigment cell and photoreceptor cell are present in the correct location, demonstrating that the eyespot forms normally.

One advantage of phototaxis is that it can enhance larval dispersal [39]. *Capitella teleta* larvae hatch from a brood tube in the sediment and are subsequently free swimming [36]. Positive phototaxis of larvae serves to bring individuals to the ocean surface [12], where they have the potential to be caught in currents that aid in dispersal [37]. Larvae of many polychaetes are positively phototactic for all, or some of their larval life [1]. More broadly, of the benthic marine invertebrates that produce pelagic larvae, the majority of these larvae have a period of positive photo response [1]. These observations emphasize the importance of light and light detection for dispersal of marine larvae to ultimately locate suitable habitat for their subsequent adult benthic life history phase.

Our results represent one of only a few published examples of CRISPR/CAS9-induced mutations causing behavioural changes in animals. In one example, mutation of the receptor for prostaglandin *F*_2∝_ prevented the initiation of sexual behaviour in the cichlid fish *Astatotilapia burton* [38]. In another example, *Opsin9* knockout disrupted oocyte maturation-inducing hormone secretion in response to light in the jellyfish *Clytia*, and prevented maturation of gonads and their subsequent release [39]. CRISPR was also used to knockout *orco* in *Harpengnathos saltator* (Indian jumping ant), dramatically affecting social and individual behaviour linked to olfaction [40].

## 4. Conclusion

Many previous studies have inferred a function for *r-opsin* in phototaxis of marine protostomes, but few have provided direct demonstration through gene mutagenesis. Our data clearly demonstrate that *Ct-r-opsin1* is sufficient to mediate positive phototaxis in *C. teleta*. Although disruption of *Ct-r-opsin1* affects larval behaviour, a morphologically normal sensory neuron of the eyespot forms. This study adds to one of very few examples using CRISPR/CAS9 technology to investigate animal behaviour, and provides mechanistic information of phototaxis in a marine larva. Analysis by genomic sequencing and *in situ* hybridization show similar high efficiency of the CRISPR/CAS9 system in *C. teleta*, and analysis of amplicon size by PCR alone is clearly an underestimate of mutation events. To our knowledge, this is the first example of CRISPR/CAS9 mutagenesis in *C. teleta*, and is among only a few examples in a spiralian*.* Our use of CRISPR/CAS9 genome editing of the *Ct-r-opsin1* gene generates an opportunity to link genotype to phenotype during post-metamorphic stages of the life cycle in future studies. Juvenile and adult worms of *C. teleta* burrow in the sediment, and we hypothesize that these stages may be negatively phototactic. Studies, such as this, expand the repertoire of functional genomic studies to a wider range of animals, and facilitate our ability to understand the evolution of animal diversity, such as in the case of the extraordinary diversity of eye structure, complexity and function in annelids.

## 5. Methods

### (a) Preparation of single guide RNA and CAS9 protein

There were 19–20 bp target sequences of candidate sgRNAs designed using CRISPRscan (www.crisprscan.org) [41] to target the *Ct-r-opsin1* coding sequence. Potential sgRNAs were manually subjected to a BLASTn search of the *C. teleta* genome (http://genome.jgi.doe.gov/Capca1/Capca1.home.html) to ensure there were no off-target hits. Three candidate sgRNAs targeting *Ct-r-opsin1* were selected (sgRNA1: GGAUGGAAGAAAGUCCCAUG; sgRNA2: GGGCUCUCCUCGAUGGGAG; sgRNA3: GAGCAGGCUAUUGCCAGCAC) and custom synthesized by Synthego (www.synthego.com). Lyophilized sgRNAs were diluted in nuclease-free 1x Tris-EDTA (TE) buffer (pH 8.0) to a concentration of 50 µM as a stock solution. Working solutions were created by dilution with nuclease-free water to a concentration of 10 µM. Both stock and working solutions were stored at −20°C. Lyophilized CAS9 protein was purchased from PNAbio (CP01–50), diluted to 2 µg µl^−1^ with nuclease-free water, and stored as single-use 1 µl aliquots at −80°C. Immediately prior to microinjection, sgRNA and CAS9 protein were mixed, and placed at room temperature for 10 min to enable formation of ribonucleoprotein (RNP) complexes. CAS9/sgRNA RNPs were then mixed with nuclease-free water and a 1 : 10 dilution of 20 mg ml^−1^ dextran (Texas Red, Molecular Probes™), before loading into needles for microinjection (see ‘Animal husbandry and microinjection' below).

### (b) Animal husbandry and microinjection

A laboratory culture of *C. teleta* was maintained following previously described methods [20]. To obtain zygotes for microinjection, females and males were first separated for 2–5 days, and then combined and checked for the presence of fertilized eggs approximately 10–12 h later. Eggs were dissected from the brood tube in 0.2 µm filtered seawater (FSW). The egg membrane was softened by a 20 s exposure to a freshly prepared 1 : 1 solution of 1 M sucrose : 0.25 M sodium citrate, followed by least three rinses in FSW. Uncleaved embryos were pressure injected using Quartz needles (QF 100–50–10) pulled on a micropipette puller (Sutter Instruments). The needles were filled with the CAS9/sgRNA mixture, a 1 : 10 dilution of 20 mg ml^−1^ fluorescent dextran (molecular probes) and nuclease-free H20. Injected and uninjected animals from the same brood were raised in FSW plus 60 µg ml^−1^ penicillin and 50 µg ml^−1^ streptomycin in separate 35 mm plastic dishes, and compared to determine the health of the brood.

### (c) *In vitro* cleavage assay

To test the ability of CAS9/sgRNA RNPs to cleave *Ct-r-opsin1 in vitro*, the following components were mixed in a 0.5 ml PCR tube to a total volume of 20 µl: 250 ng of purified *Ct-r-opsin1* PCR fragment, 250 ng (approximately 10 pmol) sgRNA, 500 ng CAS9 protein, 2 µl New England Biolabs buffer 3, 2 µl bovine serum albumin (10 mg ml^−1^). Samples were incubated at 37°C for 1 h. One microlitre RNase was added, and samples incubated for an additional 15 min at 37°C. Next, 1 µl of CAS9 stop solution (30% glycerol, 1% sodium dodecyl sulfate, 250 mM EDTA pH 8.0) was added to dissociate protein from DNA/RNA complex, and DNA fragments resulting from CRISPR/CAS9-induced cleavage were analysed by agarose gel electrophoresis.

### (d) Cloning of *Capitella teleta opsin* genes

Previous analysis identified nine *opsin* genes in the *C. teleta* genome (*opsin54244, opsin226303, opsin221903, opsin36183, opsin63256, opsin119596, opsin44169, opsin124377* and *opsin197851*) [17]. Of these, two had previously been cloned (*opsin119596,* renamed *Ct-r-opsin1* (MG225382) and *opsin197851*, renamed *Ct-n-opsin1* (MG710417)). Searches of *C. teleta* expressed sequence tags (EST) libraries (JGI, Department of Energy, Walnut Creek, CA, USA; [18]) with predicted coding sequences identified *opsin44169* (EY644637, renamed *Ct-n-opsin3*). Because the predicted coding sequence for *opsin63256* and *opsin36183* were identical, a single pair of primers was designed. Fragments of coding sequence for *opsin* genes were amplified by PCR from mixed larval stage cDNA, cloned into the pGEM-T Easy vector (Promega, Madison, WI, USA) and sequenced. Primer sequences used were as follows: *opsin54244* (F: CCTAACTTCAATCAACACACAGG; R: TTGTCGGAATCGAGGTAAGC), *opsin124377* (F: GACTTTAACTCCAGCCATACAGC; R: CAACCGGAGTCTTTTACAGC), *opsin63256/36183* (F: TGCTGGTCACGTTACTTTCG; R: ACGATTGGATTCAGACATGC), and *opsin44169* (F: GTTAGGGCTTGCAACATGC; R: GAGGAGGCCTTTAACACACC). Sequences of newly cloned, unique gene fragments (those without EST support) were submitted to the National Center for Biotechnology Information (NCBI) as original sequences with the following accession numbers: MG710415 (*opsin54244*, renamed *Ct-r-opsin2),* MG710416 (*opsin124377,* renamed *Ct-r-opsin3)* and MG710418 (*opsin63256/36183*, renamed with the single identifier, *Ct-n-opsin2)*. Cloned fragments were used as templates to generate anti-sense RNA probes for *in situ* hybridization.

### (e) Whole mount *in situ* hybridization

Following fixation in 3.7% paraformaldehyde in FSW overnight at 4°C, larvae were washed in phosphate-buffered saline (PBS), dehydrated through a methanol series to 100% methanol, and stored at −20°C for up to four weeks. Digoxigenin-labelled riboprobes were generated with either the SP6 or T7 MEGAscript kit (Ambion, Inc., Austin, TX, USA) and DIG-11-UTP (Sigma 11209256910). The following riboprobes and working concentrations were used: *Ct-r-opsin1*, 1047 bp at 0.2 ng µl^−1^ (SP6 RNA polymerase); *Ct-n-*opsin1, 865 bp at 1–3 ng µl^−1^ (T7 RNA polymerase); *Ct-r-*opsin3, 1176 bp at 1 ng µl^−1^ (T7); *Ct-n-*opsin3, 639 bp at 1 ng µl^−1^ (T7); *Ct-n-opsin2*, 722 bp at 1 ng µl^−1^ (SP6) and *Ct-r-opsin2,* 620 bp at 3 ng µl^−1^ (T7). Whole-mount *in situ* hybridization was performed following published protocols [42]. Following hybridization at 65°C for 48–72 h, probes were detected using nitro blue tetrazolium chloride/5-bromo-4-chloro-3-indolyphosphate colour substrate. The reaction was allowed to develop for 30 min −12 h depending upon the probe. *Ct-n-opsin1* was not detectable at any stages examined with 1, 2 or 3 ng µl^−1^ of probe, multiple independent repetitions, or following resynthesis of riboprobe.

### (f) Immunohistochemistry

Following *in situ* hybridization, larvae were washed several times in PBS + 0.1% Triton (PBT), then treated with block solution (PBT + 10% normal goat serum, Sigma G9023) for 45–60 min at room temperature (r.t.). The monoclonal antibody (mAb) 22C10 was diluted 1 : 10 in block solution, and animals were incubated for 2–18 h at 4°C. Animals were washed twice in PBT, followed by four PBT washes of 20**–**30 min each. Goat anti-mouse-488 secondary antibody (Invitrogen A11001) was diluted 1 : 250 in block solution, and incubated with animals for 2–4 h at r.t., followed by two rinses in PBT, and four PBT washes of 20**–**30 min each prior to analysis. The mAb 22C10 was deposited to the Developmental Studies Hybridoma Bank by Benzer, S./Colley, N. (DSHB, Department of Biology, University of Iowa, Iowa City, IA, USA).

### (g) Microscopy and imaging

Following *in situ* hybridization, larvae were imaged using an Axioskop 2 motplus compound microscope (Zeiss, Gottingen, Germany), coupled with a SPOT FLEX digital camera (Diagnostic Instruments, Inc., SterlingHeights, MI). Images were captured using SPOT imaging software and analysed using Adobe Photoshop CS6 (v. 13.0). Multiple differential interference contrast microscopy focal planes were merged for some images using Helicon Focus (Helicon Soft Ltd., Kharkov, Ukraine), as noted in figure legends. Following immunohistochemistry, larvae were imaged using a Zeiss LSM 710 confocal microscope (Zeiss, Gottingen, Germany). Z-stack projections were generated using Fiji [43]. All figures were created in Adobe Photoshop CS6 (v. 1.3.0), or Adobe Illustrator CS6 (v. 16.0).

### (h) Analysis of CRISPR/CAS9-induced genomic editing

Genomic DNA extraction buffer (0.01 M Tris pH8.0, 0.05 M KCl, 0.3% Tween-20, 0.3% NP-40, 0.001 M EDTA, 0.5 mg ml^−1^ proteinase K) was freshly prepared and placed on ice. Single larvae were placed on the inside of a lid of a 0.5 ml PCR tube, as much seawater removed as possible, and then 20 µl of extraction buffer was pipetted onto the larva. Tubes were centrifuged briefly to bring larva/buffer to the tube bottom, vortexed, briefly spun again, and then placed at 55°C for 2–3 h. Tubes were vortexed every 30 min during incubation. Next, proteinase K was inactivated by incubation at 98°C for 5 min. PCR was conducted using 5 µl of gDNA as input template with ExTaq DNA polymerase (Takara, RR001A) and *Ct-r-opsin1* specific primers (F: 5′ TAACTGGCATGGCATACACG; R: 5′ TTGGATTCCACATAGCAGAGG). Cycling conditions were as follows: initial denaturation, 95°C, 2 min; 35 cycles (95°C, 30 s; 56°C, 30 s; 72°C, 2 min); final extension, 72°C, 5 min. Resulting fragments were analysed by agarose gel electrophoresis.

### (i) Phototactic assay and statistical analyses

Phototactic behaviour was assessed using a custom-built chamber based upon the design described in [2]. The chamber consists of a black plastic box, with slits at each end for removable shutters. A diffuser made of sandblasted glass was covered in black electrical tape to restrict light entry to a 5 mm width vertical sliver of glass. Up to 10 larvae were placed in a glass square cuvette (15 × 15 × 4 mm), which was covered on all sides with black electrical tape, aside from a 7.5 mm sliver on one side. A white LED light was positioned to one side of the chamber, and the glass cuvette containing larvae placed within the chamber, orienting the uncovered sliver towards the light source. Larvae were imaged from above by transmitted light that passed through an infrared filter (X-Nite780, LDP LLC). Larvae were dark adapted for at least 1 min, and then the shutter closest to the external light source was removed for 1 min. All behavioural assays were filmed using a xiQ camera, with a frame rate of 90 frames s^−1^ (MQ042CG-CM; Ximea). Positional information for each larva was recorded 20 s after initial light exposure. The cuvette was divided into four quadrants: quadrant 1 (*Q*1) nearest the light source, and the remaining quadrants termed quadrants 2, 3 and 4 (*Q*2–4). Larvae in *Q*1 at 20 s were scored as ‘near’ the light source (positive phototaxis), and larvae in *Q*2–4 were added together and scored as ‘far’ from the light source (no phototaxis). Statistical analysis (Fisher's one-tailed exact test) was performed using GraphPad Quick Calcs (http://www.graphpad.com/quickcalcs/).

## Supplementary Material

Table 1

## Supplementary Material

Video of C. teleta larval phototaxis

## Data accessibility

Data concerning CRISPR/Cas9 mutagenesis conditions and scoring are provided in the electronic supplementary material, table S1. Sequences of cloned opsin genes have been deposited to NCBI and accession numbers are listed in the Methods. Detailed *in situ* hybridization protocol is available on the Seaver laboratory website at https://www.whitney.ufl.edu/people/current-research-faculty/elaine-c-seaver-phd/protocols/
