# Supplementary material for: CRISPR/CAS9 mutagenesis of a single r-opsin gene blocks phototaxis in a marine larva
Source: Proc Biol Sci. 2019 Jun 5;286(1904):20182491. doi: 10.1098/rspb.2018.2491 (PMC6571462; doi:10.1098/rspb.2018.2491)
Supplement: Table 1 [file rspb20182491supp1.docx]

|  | **sgRNA concentration** | **CAS9 concentration** | **Molar ratio (CAS9:sgRNA)** | **Phototaxis Assay** | | | **Wildtype transcript by ISH**  **(4 eye spots)** | **% Larvae with expected amplicon size (PCR analysis)** | **% Larvae with wildtype genotype (sequence analysis)** |
| --- | --- | --- | --- | --- | --- | --- | --- | --- | --- |
|  |  |  |  | **Q1**  **(n=)** | **Q2+Q3+Q4**  **(n=)** | **Statistical signif. (vs CAS9 only)** |  |  |  |
| **A** | 3.6 pmol/μL | 3.7 pmol/μL | 1:1 | 24 | 38 | * (p=0.0273) | 84% (n=77/92) |  |  |
| **B** | 1.7 pmol/μL | 2 pmol/μL | 1.2:1 | 17 | 49 | **  (p=0.0002) | 0%  (n=0/25) | 35% (n=12/34) | 0%  (n=0/9)^a^ |
| **C** | 3 pmol/μL | 5 pmol/μL | 1.7:1 | 18 | 47 | **  (p=0.0005) | 4%  (n=2/49) | 31% (n=4/13) |  |
| **sgRNA only** | 1.7 pmol/μL |  |  | 44 | 38 | n.s.  (p=0.4175) | 90%  (n=45/50) | 100% (n=10/10) |  |
| **CAS9 only** |  | 2 pmol/μL |  | 43 | 33 | - | 78%  (n=47/60) | 100% (n=24/24) |  |

**S2 Table 1. Differences in efficiency of genome editing associated with different ratios of sgRNA to CAS9 protein**

(^a^: Genomic DNA from 9 single larvae was extracted and PCR using *Ct-r-opsin1*-specific primers conducted. 5 larvae had mutant sized bands extracted and cloned, and 1-5 clones of each were sequenced. 4 larvae had WT sized bands extracted and cloned, and 4-8 clones of each were sequenced. Each clone from the 5 larvae contained large deletions. Clones from the 4 larvae consisted of either a mix of WT and mutant sequences (n=1), or all clones had small insertions and/or deletions (n=4)). Q, quadrant.
